# Tai Chi training as a primary care plan for the prevention and management of hypertension: an opinion and positioning article

**DOI:** 10.1080/07853890.2024.2320863

**Published:** 2024-02-19

**Authors:** Ting Zhang, Shuman Yang, Wei Liu, Qingping Bai, Song Gao

**Affiliations:** a College of Physical Education and Health Sciences, Zhejiang Normal University, Jinhua, China; b University Hospital, Zhejiang Normal University, Jinhua, China; c Physical Education College, Guangxi University of Science and Technology, Liuzhou, China

**Keywords:** Hypertension, exercise therapy, Tai Chi, prevention and management, limitations

## Abstract

Hypertension is a prevalent chronic condition worldwide that can impact patients’ quality of life. Oral antihypertensive drugs are widely used to manage high blood pressure, primarily by regulating the renin-angiotensin-aldosterone system. Nevertheless, limited efficacy and low compliance represent significant obstacles, arising primarily from dose, duration, and medication type restrictions. Furthermore, the prolonged use of antihypertensive medication may result in dependence and adverse effects, without any substantial improvement in achieving targeted blood pressure leves. As a result, research has focused on using exercise therapy to treat hypertension. Tai Chi, a widely-practiced Chinese health exercise, has evolved into a form of exercise therapy that might help alleviate the risk associated with hypertension. Therefore, this article aims to outline the role of Tai Chi in preventing and managing hypertension.

## Introduction

Hypertension, defined as a clinical blood pressure ≥140/90 mmHg, is a common chronic disease with the highest prevalence worldwide [1]. Hypertension causes headaches, dizziness, palpitations, numbness in the limbs and can even cause cerebral hemorrhage [2]. Its pathological basis is water and sodium retention or endothelial damage caused by various factors, and it is a risk factor for obesity, metabolic syndrome, dyslipidemia, and stroke [3,4]. Globally, 31.1% of adults (1.39 billion people) have hypertension [5]. However, changes in the prevalence of hypertension have not been uniform throughout the world. Over the past two decades, the prevalence of hypertension has declined slightly in high-income countries, while it has increased substantially in low- and middle-income countries [5]. The China Hypertension Survey (CHS) showed that in 2015, the crude prevalence of hypertension among adults aged ≥18 years in China was 27.9%, with a total prevalence of approximately 244.5 million [6]. Epidemiology shows that there are 80million hypertensive patients aged ≥ 20 years in the U.S. Hypertension is also associated with gender, with more men than women having hypertension aged 45–65 years, and more women than men having hypertension aged 65 ≥ years [7].

Oral antihypertensive medications are the most common treatment for high blood pressure. Medicines are mainly used to treat hypertension by regulating the dysfunction of the renin-angiotensin-aldosterone system [8]. However, compliance and effective control of hypertension are not high due to limitations in the dose, duration, and type of medication taken. In addition, long-term use of antihypertensive drugs can lead to dependence and other side effects, with no significant effect on associated symptoms such as chest tightness and palpitations [9,10]. Therefore, studies have attempted to improve hypertension with exercise therapy [11,12]. Exercise therapy has significant advantages in this regard, with significant efficacy and low drug dependence. In the early twentieth century, the WHO and the International Society of Hypertension revised the guidelines for the treatment of hypertension to include exercise as one of the forms of antihypertensive therapy [13]. Long-term cardiorespiratory endurance training is characterized by low intensity, long duration, and high rhythm, which may be effective in controlling hypertension [14]. Studies have shown that resistance training lowers blood pressure as much or more than aerobic training [15,16]. However, resistance training involves more forceful movements and patients may experience reactions such as dizziness, nausea and stomach upset, so a pre-assessment of the participant’s physical condition is required [17].

The ACSM statement notes that complementary or alternative types of neurological exercise, such as yoga, Pilates and Tai Chi, have been shown to lower blood pressure [18]. Some studies have concluded that moderate-intensity aerobic exercise is superior to low-intensity and sustained high-intensity exercise in preventing and reducing blood pressure [19]. It is important to note that the classification of exercise intensity is an artificially defined range, and there are individual differences. Aerobic exercise has been found to produce more endogenous β-endorphins, which in turn reduces the hyperexcitability of the sympathetic nervous system [20,21]. Long-term exercise improves vascular endothelial function and reduces plasma endothelin levels to relieve the highly constricted state of blood vessels [22,23]. Exercise can block the over-activation of the renin-angiotensin-aldosterone system, reduce plasma renin activity, angiotensin II, and aldosterone plasma levels, and improve vasodilatation [24].

Tai Chi is a light-to-moderate intensity mind-body exercise worthy of long-term practice, and its safe and effective features have attracted the attention of the public and researchers [25]. A single session of aerobic exercise can produce a short-term antihypertensive effect (i.e. lasting from a few hours to 1 d) in both normal subjects and hypertensive patients, a phenomenon known as exercise hypotension [26]. A study by Bersaoui et al. showed that long-term moderate-intensity aerobic exercise improved blood pressure levels in an Asian population, with mean systolic and diastolic blood pressure decreasing by 7.2 and 4.7 mmHg, respectively [27]. Another study found that after 10 weeks of regular exercise in elderly hypertensive patients, systolic and diastolic blood pressure levels were reduced by 13 mmHg (1 mmHg = 0.133 kPa) and 18 mmHg, respectively, but the antihypertensive effect was not enhanced when the frequency of exercise was greater than three times a week [28]. A meta-analysis concluded that swimming training at moderate intensity, 3 times/week for 8–11 weeks had the most significant effect on systolic blood pressure reduction among all exercise types, frequencies and duration groups [29]. The cost of Tai Chi training is about $3.50 per class, which is not a financial burden for participants [30]. People of different ages and physical conditions can easily perform Tai Chi training, and only a common place is needed. Compared with conventional aerobic exercise, Tai Chi training does not require special equipment and venues, and the intensity and difficulty of the exercise are not too high, which makes it easy for patients with hypertensive to practice.

The quality of life of patients with hypertension is threatened, and there is an urgent need to find safe and effective complementary and alternative therapies. Tai Chi plays an important role in the daily management of chronic diseases, and its value in the prevention and treatment of hypertension deserves further investigation. Therefore, this paper aims to summarise the clinical research results of Tai Chi in improving hypertension, analyse the limitations and challenges of current research, and propose a new direction.

## Tai Chi for persons with hypertension

High blood pressure can lead to cardiovascular diseases such as cerebral hemorrhage, stroke, and heart attack [31]. If a patient is diagnosed with hypertension, he or she needs to adhere to antihypertensive medication and good lifestyle habits, such as a low-salt, low-fat diet [32]. In addition, the search for active and effective exercise to complement the treatment of hypertension is particularly important to improve the quality of life of patients with hypertension. At present, most studies have confirmed the clear effect of Tai Chi on lowering blood pressure, improving blood lipids and quality of life in hypertensive patients [33–35]. The characteristics of the included studies are shown in Table 1.

**Table 1.** Characteristics of the included studies.

Author	Sample (mean age)	Gender(M/F)	Intervention	Frequency (per week) and duration	Control group	Measured outcomes
Ma C 2018 (China) [36]	TC: 79 (70.24 ± 10.25) C:79 (69.71 ± 10.84)	TC: 54/25 C: 55/24	24-style Tai Chi + usual care	1h, 3–5x, 24w	Usual care	SBP*, DBP*, BMI*, Waist girth
Sun J 2015 (AUS) [38]	TC: 136 C: 130	TC: 19/117 C: 29/101	Tai Chi	3 h/w and 2h practice by themselves at home,1year	None exercise-related activities such as reading and learning computer software applications	SBP*, DBP*, BMI*, HRQoL
Lo HM 2012 (China) [37]	TC: 27 C: 31	/	Yang-style Tai Chi	1h, 3x, 8w	Usual care	Routine Health Care Behaviour scal; BP
Shou XL 2019 (China) [39]	TC:104 C: 104	/	24-Style Simplified Tai Chi	20–30min, 2x /day, 3 months	General daily lifestyle intervention	SBP*, DBP*, BMI*, PP, TC, TG, and LDL-C
McDermott K 2015 (USA) [40]	Chi-Running: 10 (56.4 ± 8.2) C:12 (54.1 ± 4.5)	CR: 5/5 C: 6/6	Chi-Running	4h at week 0, and three more times for 2h training at weeks 2, 4, and 8.	Active control/self-directed control	SBP, DBP, BMI*; injure
Tsai JC 2003 (China) [34]	TC: 37 (51.6 ± 16.3) C: 39 (50.5 ± 9.8)	TC: 19/18 C: 19/20	Tai Chi	30min, 3x, 12w; 64% of maximal heart rate	/	BP*, lipid profile* and anxiety status*
Pan X 2015 (China) [43]	TC: 24 (51.6 ± 16.3) HP: 16 (50.5 ± 9.8)	/	Tai Chi	1h, 6x, 12w	Usual care	blood glucose, cholesterol, NO*, CO*, H_2_S* and BP*

***Abbreviations***: SBP, Systolic blood pressure; DBP, Diastolic blood pressure; BMI, Body Mass Index; HRQoL, Health-related quality of life; BP, Blood pressure; PP, Pulse pressure; TC, Total cholesterol; TG, Triglyceride; LDL-C, Low-density lipoprotein cholesterol. *indicates statistically significant differences between the intervention group and the control group (*p* < 0.05).

Persons with hypertension can not only lower their blood pressure during Tai Chi training, but also improve their quality of life by promoting general health [35,36]. An 8-week Yang-style Tai Chi training by Lo et al. confirmed that Tai Chi can improve exercise performance and time in patients with hypertension [37]. A study by Ma et al. [36] reported that 24 weeks of group-based Tai chi was effective for elderly patients with hypertension. Sun et al. [38] conducted a long-term Tai Chi intervention, and they randomly divided community-dwelling older adults with hypertension into the Mind-Body Meditative Tai Chi Program group and the control group (such as reading and learning software applications). It was found that after a long period, the Tai Chi group markedly improved SBP, DBP, BMI, epithelial growth factor receptor (eGFR), physical health scales (including role of physical health, bodily pain, and total physical health of HRQoL), and the vitality scale of mental health HRQoL. However, there were no significant changes in any of the outcome measures in the control group. This provides evidence for the community application of Tai Chi to improve blood pressure and quality of life in patients with hypertension.

Focusing on patients with hypertension among young and middle-aged adults, Shou et al. [39] conducted a 3-month Tai Chi intervention and compared the effectiveness of Tai Chi training for 1 month and 3 months. The study showed that SBP, heart rate (HR), triglycerides (TG), total cholesterol (TC), and low-density lipoprotein cholesterol (LDL-C) all changed significantly after 1 month, while BMI, blood glucose (Glu), DBP and pulse pressure (PP) did not change significantly. After 3 months of Tai Chi training, BMI, HR, SBP, DBP, PP, TG, TC, LDL-C, and Glu all changed significantly. This is consistent with a study of the effects of Tai Chi on blood lipid changes in patients with hypertension [34]. Furthermore, there is a pre-experimental study on Tai Chi running, which shows that Tai Chi running is an acceptable exercise for patients with hypertension and has a lowering effect on BMI [40].

At present, most studies have confirmed that Tai Chi has good effects on reducing blood pressure, improving blood lipids and quality of life in patients with hypertension, but the mechanism is unclear [41,42]. The research by Pan et al. [43] found that the vasodilatory endogenous gas signalling molecules such as NO, CO and H_2_S were altered in patients with hypertension. Tai Chi, as a traditional Chinese exercise, has fewer side effects, lowers lipids, lowers glucose, lowers C-reactive protein (CRP) and other inflammatory factor concentrations, and is useful in protecting vascular endothelial function and delaying the onset of hypertension. More research is needed on the mechanism of blood pressure lowering by Tai Chi.

## Limitations of current Tai Chi research

Although traditional Chinese exercise therapy, as represented by Tai Chi, has shown good effects in improving hypertension, there are some limitations to the studies. At present, the vast majority current controlled clinical trials have recruited subjects without any basic Tai Chi training; Tai Chi training has generally not exceeded 6 months to assess differences from the control group [44,45]. Thus, this may result in different research findings. There is a lack of large, multicenter trials of Tai Chi for hypertension. At present, many studies have only examined its effects on blood glucose and lipid metabolism and blood pressure, ignoring the damage to the vascular endothelium by the inflammatory response, and relevant effect indicators should be added in future studies to improve the strength of the evidence. Nocturnal blood pressure and circadian rhythms are important factors in good blood pressure control. However, current studies have only confirmed that Tai Chi improves circadian rhythms in patients with insomnia [46]. In addition, Tai Chi protocols for improving hypertension are not standardized, and the duration and frequency used in different studies are inconsistent. It is recommended that standard exercise protocols that are consistent with disease progression be explored to improve the reproducibility and generalizability of study results. Hypertension occurs at different ages and in different conditions [47], and there are fewer trials of traditional exercise therapy across age groups and conditions (e.g. prehypertension, H-type hypertension), which is an important area for future research.

Tai Chi, as a light-to-moderate intensity aerobic exercise, can be practiced three times a week to meet the standards recommended by the European Society of Cardiology (ESC) guidelines for physical activity in patients with sports cardiology and cardiovascular disease [48,49]. Patients with hypertension are recommended to do at least 30 min/day of moderate-intensity aerobic exercise (walking, jogging, cycling, or swimming) for 5–7 days/week [50]. Despite the above relevant guideline recommendations, there is no fixed approach to how to exercise specifically for each case of hypertension, and it is important to consider both evidence-based medical evidence and to vary from person to person and place to place to achieve the maximum benefit-to-risk ratio. Besides, Tai chi is a mind-body exercise, and it can be combined with breathing exercises and soothing music to effectively improve the negative mood in people with high blood pressure [34,49].

## Conclusion

The author points out that Tai Chi can improve hypertension and is a safe and effective non-drug intervention. However, there are different forms of Tai Chi and none of the styles are specifically designed for hypertension. The complexity of Tai Chi hinders its use in patients with hypertension. Therefore, it is urgent to develop a series of simplified Tai Chi suitable for hypertensive patients with different levels of physical activity. Further research is needed to understand the mind-body benefits of Tai Chi as a multi-component exercise or as an individual treatment for hypertension. Although Tai Chi has potential, its therapeutic effects need to be further investigated because the mechanism of intervention is still unclear. The mechanism by which Tai Chi ameliorates hypertension is a key issue to be considered in future research.

## Data Availability

Data sharing is not applicable to this article, given that no new data were created or analysed in this study.
